# A novel Adolescent Health Behavior Checklist

**DOI:** 10.3389/fpubh.2025.1438775

**Published:** 2025-02-25

**Authors:** Yanjie Su, Hua Bai, Ying Li, Yang Zhang

**Affiliations:** ^1^School of Sport Education, Tianjin University of Sport, Tianjin, China; ^2^College of Physical Education, Xingtai University, Xingtai, China; ^3^Independent Researcher, Windermere, FL, United States

**Keywords:** mental health, health literacy, physical activity, sleep, nutrition

## Abstract

**Purpose:**

Adolescents are experiencing rising rates of obesity, insufficient exercise, and sleep disorders. To provide a scientific basis for policymakers to develop targeted and evidence-based health behavior education and policies, this study employed structural equation modeling to design the Adolescent Health Behavior Checklist (AHBC).

**Methods:**

We designed a draft 6-dimensional AHBC, which includes the dimensions of exercise, diet, personal responsibility, sleep, interpersonal relationships, and stress management. Each item is rated on a 5-point Likert scale, with higher scores indicating healthier behavior. Through exploratory factor analysis (EFA) and confirmatory factor analysis (CFA), we optimized the construct validity of the AHBC.

**Results:**

The optimal factor structure was first determined using EFA with 177 middle school students participating in the process. EFA suggested a hierarchical, 6-factor AHBC with good internal consistency (global Cronbach's alpha = 0.96). Using an independent sample of 349 middle school students, CFA confirmed the construct validity of the AHBC. The final model demonstrated a good fit: SRMR = 0.058, CFI = 0.990. Five out of six latent variables had factor loadings higher than 0.7, and 81% of the item-level factor loadings exceeded 0.7. Additionally, all latent variables had McDonald's omega values higher than 0.7, indicating acceptable convergent validity. Finally, factor correlations showed that the AHBC has good discriminant validity.

**Conclusions:**

The AHBC is a 31-item checklist that assesses adolescents' all-around health behaviors, using a score of four as the benchmark value. The shortcomings of the current checklist are discussed, along with future theoretical and practical directions for improvement.

## 1 Introduction

The healthy growth of adolescents is the cornerstone of societal progress. Nevertheless, the course of modernization has brought forth various factors that adversely impact this growth. Globally, the development of national economies has exacerbated the issue of adolescent obesity (1), driven by the widespread consumption of highly processed foods and a lack of healthy dietary principles (2). Adolescents addicted to digital media, such as social media and video games, are less inclined to participate in daily exercise (3). This lack of physical activity during this crucial period of habit development might lead to significant health consequences in the long run. Additionally, academic and social pressures have increased the occurrence of chronic anxiety among adolescents, often leading to sleep disorders that affect both physical and mental wellbeing (4, 5). Moreover, the problem of school bullying (6, 7), which goes against social morality, underscores the need to promote prosocial behaviors among adolescents. These challenges highlight the urgent need to foster all-around health behaviors in adolescents in the context of modernization. Therefore, designing an instrument that can objectively measure adolescent health behaviors is of great practical importance.

It is useful to establish a clear definition of health first. The conventional notion of health, which defines it solely as the absence of illness, is no longer applicable in modern society. For example, obesity is widely recognized as a precursor to metabolic diseases, making it a consequence of unhealthy behaviors (e.g., poor diet choice, and inadequate exercise) and a significant risk factor for more serious conditions. Therefore, being overweight is a subclinical state from a metabolic health perspective (8). Similarly, social skills deficit not only hampers the overall development of adolescents but can also lead to the formation of antisocial thoughts, which may result in more severe consequences (9). Therefore, the modern concept of health is a multidimensional corollary of physical, mental, and prosocial development. According to the WHO, health is a state of complete physical, mental, and social wellbeing.

Although there is a consensus on the meaning of health, the system for assessing health behaviors has not yet been perfected. In China, there is no widely used health behaviors assessment scale for adolescents, which hinders the assessment of Chinese adolescents' health behaviors. Currently, only three studies have explored the design of health behavior assessment instruments for this demographic. Zhang and Tang used exercise habits, emotional regulation, and adaptability as dimensions of health behaviors among middle school students (10). Zhao and Xu categorized middle school students' health behaviors into five dimensions: awareness of physical activity, exercise habits, health knowledge, emotional control, and environmental adaptation (11). Guo constructed a health behavior scale for high school students, assessing four dimensions: physical activity awareness and habits, health knowledge acquisition and use, emotional control, and environmental adaptation (12). Although these studies have initially filled the gap in health behavior assessment among Chinese adolescents, the scales remain relatively narrow. For instance, none of the measures evaluate sleep behavior and eating behavior, both of which are becoming increasingly crucial for adolescents' wellbeing (13, 14).

There are currently four generally recognized instruments for assessing adolescent health behaviors. First, the Global School Health Policies and Practices Survey (15), endorsed by the WHO, is the most authoritative and comprehensive instrument currently available. The questionnaire assesses a total of 128 items across five dimensions: healthy and safe school environment, health services, nutrition services, health education, and physical education. Although originally intended for assessing school health policy, its comprehensiveness is valuable for relevant research. Second, while the Health-Promoting Lifestyle Profile was originally designed for adults (16), previous research has shown that this instrument can also evaluate the health behaviors of adolescents (17). Two Chinese-language simplified versions of the instrument have been validated (18, 19). This instrument assesses self-actualization, health responsibility, exercise, nutrition, interpersonal support, and stress management, which reflects the commonly accepted dimensions of health. Third, Chen et al. created the Adolescent Health Promotion scale, an instrument that assesses six dimensions of health behaviors: social support, life appreciation, health responsibility, dietary behaviors, exercise behaviors, and stress management (20, 21). Fourth, the Youth Risk Behavior Surveillance System is a self-administered questionnaire designed to assess high-risk behaviors among high school students. Since its inception, this instrument has become the largest public health surveillance system in the United States. The existing scales not only cover exercise, nutrition and diet, interpersonal relationships, and stress management, but also focus on the distinct development of adolescent psychological maturation and assess personal responsibility (i.e., high-risk behaviors), which are of great value. However, these instruments can be improved, particularly as they do not assess the growing occurrence of abnormal sleep patterns in adolescents.

To enhance the overall wellbeing of adolescents, the Chinese government has implemented several national school health initiatives in recent years (22–24). Nonetheless, the lack of a comprehensive health behavior assessment instrument specifically designed for adolescents hinders policymakers from accurately assessing the progress of these policies. Therefore, the purpose of this study was to design a novel Adolescent Health Behavior Checklist (AHBC). The AHBC incorporates six key dimensions relevant to adolescent wellbeing: exercise, diet, personal responsibility, sleep, interpersonal relationships, and stress management. The ultimate objective is to ensure a scientific evaluation of adolescents' health behaviors, both in China and globally.

## 2 Methods

### 2.1 Generation of draft AHBC

**Figure 1:**
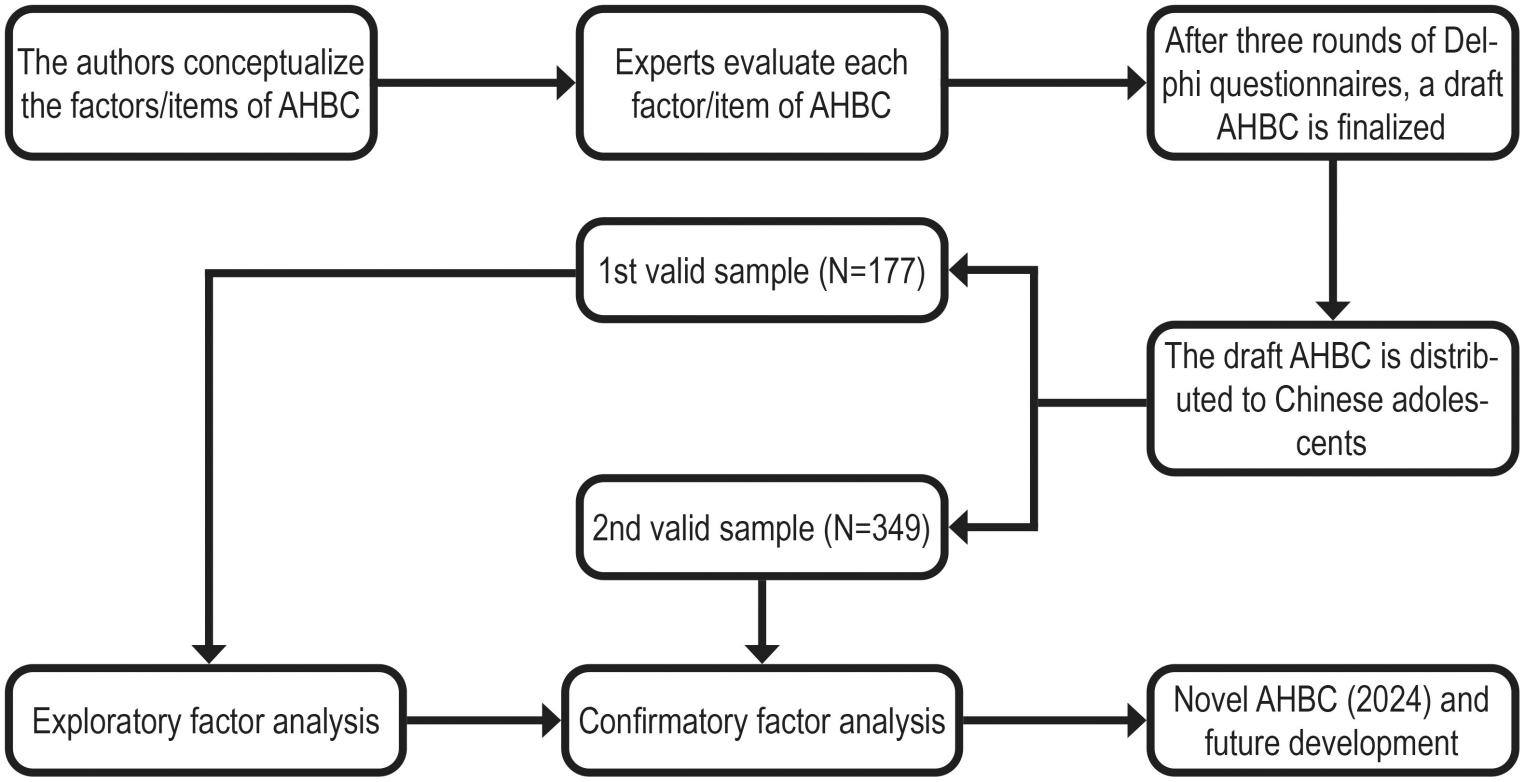
Flow chart of the Adolescent Health Behavior Checklist (AHBC) design process.

Figure 1 illustrates the design process of the AHBC. The draft AHBC was achieved through a comprehensive literature review combined with the Delphi method. Initially, based on existing instruments (16–21, 25, 26), we conceptualized an exhaustive list of potential theoretical items for each of the six factors. Adolescents' exercise behavior dimensions include the frequency and intensity of exercise, both before and after exercise, exercise awareness, and exercise habits. Diet behavior dimensions encompass snacking, eating habits, and eating perceptions. Personal responsibility behavior dimensions cover alcohol abuse, smoking, behavioral addictions, accident avoidance, safety education and skills, safe sex behavior, health hygiene habits, regular health checkups, rational medication use, and timely access to medical care. Sleep behavior dimensions consist of sleep disorders, sleep quality, sleep duration, and sleep phase. Interpersonal relationship dimensions involve managing social networks, integrating into groups, cooperation, and communication skills. Finally, stress management behavior dimensions include stressors, attitudes toward stress, stress relief methods, and emotional control. The evaluation is based on a 5-point Likert scale, where one represents never, two represents rarely, three represents sometimes, four represents often, and five represents always.

The Delphi method is a questionnaire-based approach in which the questionnaire creator and field experts collaborate over several rounds of discussion and deliberation to reach a consensus. We invited 12 experts to evaluate a preliminary list of AHBC items. All these experts hold doctoral degrees and have at least 5 years of professional experience in adolescent psychology, physical activity, health promotion, or sports pedagogy. We conducted three rounds of Delphi interviews. Experts provided written informed consent to participate (see also Ethics Approval below). The first round involved face-to-face inquiries regarding the dimensions and scale of the AHBC. Initial items that received disagreements from experts were either excluded or revised during the second round of email questionnaires. All experts agreed on the draft AHBC after the third round of email questionnaires. As a result, a total of 40 items were compiled for the draft AHBC.

### 2.2 Sampling

This study was approved by the Ethics Committee of Tianjin University of Sport (approval #: TJUS 2023-044). Two convenient samples were randomly drawn from three middle schools in Tianjin. Participants were informed that their participation in the research was voluntary, and their legal guardian provided written informed consent. For the exploratory factor analysis (see below), the sample size was determined using a 5:1 participant-to-item ratio. For the confirmatory factor analysis, the sample size was determined using a 10:1 participant-to-item ratio. Volunteers participated in either the exploratory factor analysis portion of the survey or the confirmatory factor analysis portion. All surveys were administered in classrooms using paper and pencil. If participants had any questions, researchers were available on-site to provide assistance.

### 2.3 Statistics

We conducted an exploratory factor analysis to determine the optimal factor structure of the AHBC, and subsequently, we employed confirmatory factor analysis to validate this structure. Statistical analyses were carried out using R (version 4.3.1) with packages including lavaan (version 0.6-17), psych (version 2.4.3), semTools (version 0.5-6), and semPlot (version 1.1.6).

First, we evaluated the factorability of the AHBC items using the Kaiser-Meyer-Olkin (KMO) measure and Bartlett's test of sphericity. The KMO test assesses the partial correlations among item pairs, with a KMO value exceeding 0.80 indicating adequate sampling. Bartlett's test, on the other hand, tests the null hypothesis that bivariate correlations among items are zero; significant results suggest the suitability of the item correlation matrix for factor analysis.

Second, we chose principal axis factoring as the extraction method for the exploratory factor analysis. Initially, we performed a parallel analysis to ascertain the expected number of common factors. Subsequently, factors were extracted using weighted least squares estimation with an oblimin transformation. Items were retained if their communality exceeded 0.4, they exhibited oblique factor loadings of at least 0.5 on their primary factor, and showed no or cross-loadings below 0.2 (27). Furthermore, we used Cronbach's alpha to assess the internal consistency of the extracted factor structure. A Cronbach's alpha value higher than 0.7 indicates acceptable internal consistency.

Third, the revised draft of the AHBC underwent validation with an independent sample. Confirmatory factor analysis assumes multivariate normality, a statistical assumption sometimes achieved by logarithmic transformation of raw data or using distributional alternatives (28). However, statistical advancements advocate for using diagonally weighted least squares estimation for ordinal data from Likert-type scales, as it yields more reliable results (i.e., factor loadings) compared to robust maximum likelihood estimation (29). Hence, we utilized diagonally weighted least squares estimation in this study for confirmatory factor analysis.

Rather than relying on multiple fixed fit indices, we adopted a modern statistical approach that focuses on fewer but more accurate indices to assess model fit (30). Specifically, we prioritized the standardized root mean residual (SRMR), which demonstrated an exceptional hit rate (94.4%) of model fit in simulations, with a benchmark value set at 0.6 instead of the traditional 0.8 level (31). This stricter criterion ensured a more rigorous evaluation of model fit. Additionally, we reported the comparative fit index (CFI) alongside SRMR for future comparative analyses.

After confirming the model's fit, we proceeded to assess the reliability, convergent validity, and discriminant validity of the AHBC. McDonald's omega was calculated to measure reliability, while factor loadings and average variance extracted (AVE) were used to evaluate convergent validity. If an item's factor loading fell below 0.5, no *post hoc* modifications (i.e., allowing correlated error terms) were performed. Instead, the item was directly excluded, and the model was refitted accordingly. If McDonald's omega exceeded 0.7, all factor loadings were above 0.5, and/or AVE values surpassed 0.5, we considered reliability and convergent validity achieved (32).

For assessing discriminant validity, we employed Rönkkö and Cho's factor correlation method, scaling latent variables by fixing their variances to one [i.e., setting model fit value for std.lv as TRUE in lavaan; (33)]. At a cutoff of 0.8 for the constrained model, if both the point estimate and its 95% upper limit were below 0.8, or if likelihood ratio tests were significant at the 0.05 level, this indicated evidence of discriminant validity.

## 3 Results

### 3.1 Exploratory factor analysis

Given that the draft AHBC contained 40 items, we distributed 200 questionnaires, of which 177 were valid for analysis. The KMO test value was 0.90, indicating very small partial correlations. Bartlett's test showed that the matrix had non-zero bivariate correlations, χ^2^ (561) = 3,840, *p* < 0.001. These results suggest a favorable matrix for exploratory factor analysis.

**Figure 2:**
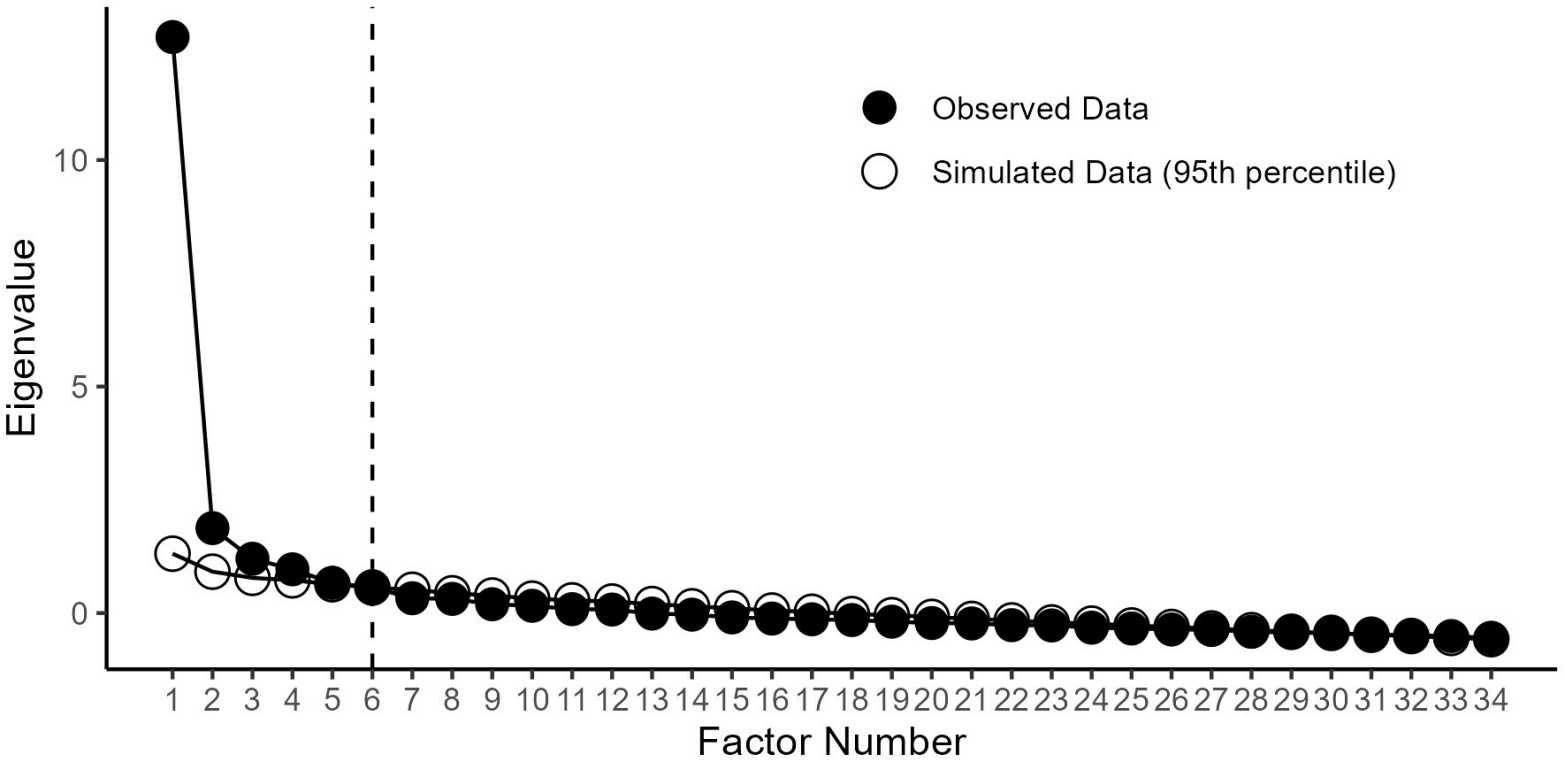
Scree plot of the exploratory Adolescent Health Behavior Checklist.

The results of the parallel analysis are visualized in Figure 2. Based on a 6-factor structure, we performed three rounds of principal axis factor analysis with oblique rotation, resulting in the elimination of six items. The exploratory AHBC is presented in Table 1, and its factor structure is shown in Table 2. Item communalities ranged from 0.48 to 0.80, indicating that a moderate proportion of the variance in each item is explained by the 6-factor structure. In total, these six factors accounted for 64% of the item response variance. As a final step of exploratory factor analysis, the draft AHBC exhibited acceptable internal consistency at both the global and factor-item levels (Table 3).

**Table 1:** Adolescent health behavior checklist.

**A. exercise**
A1. You warm up before engaging in any strenuous exercise.
A2. You do cool down after any strenuous exercise.
A3. Despite your heavy study load, you have been able to maintain your physical activity.
A4. You can perform high-intensity, approximately 30-min exercises (excluding warm-up) at least three times a week.
A5. You wait at least half an hour after your meal before exercising.
A6. You will wear protective gear during your workout to prevent injuries from overexertion.
**B. diet**
B1. You eat breakfast every day and have three regular meals daily.
B2. You can focus entirely on eating when you have your meals.
B3. You prefer chewing slowly when you eat.
B4. You make conscious choices to opt for less salty foods.
B5. You pay more attention to achieving a balanced mix of different foods when you eat.
B6. You are interested in learning more about healthy eating.
**C. personal responsibility**
C1. You have strong willpower and exhibit no signs of mentally addictive behaviors.
C2. You are aware of road safety, electrocution hazards, and drowning safety to prevent incidents.
C3. You continuously acquire health and safety knowledge and skills over time.
C4. You protect your eyes by employing various methods, including eye exercises, using eye drops, and avoiding excessive eye strain.
C5. You maintain good health and hygiene practices, such as washing your hands before meals and brushing your teeth in the morning and evening.
C6. You have learned about the characteristics of adolescent puberty from various sources, including the Internet and public information.
**D. sleep**
D1. You don't have a sleep disorder and can fall asleep quickly.
D2. You wake up feeling energized and refreshed.
D3. You believe that you've had good quality sleep in the last month.
D4. You get a full 8 h of sleep each day.
D5. You have consistently been able to fall asleep by 22:30 every day in the last month.
**E. interpersonal relationships**
E1. You possess excellent interpersonal skills.
E2. You remain close to the people you care about and show genuine concern for their wellbeing.
E3. You take the initiative to meet students from different classes, expanding your social interactions.
E4. You have good integration skills and can quickly adapt to and become part of any group.
E5. You possess strong cooperation skills and can effectively collaborate with classmates or partners to achieve shared goals.
E6. You regularly reach out to your loved ones to express care and concern.
F1. You can identify the source of stress whenever you experience it.
F2. You bravely accept the things in your life that you cannot change.
F3. You handle difficulties and setbacks with resilience and composure.
F4. You actively seek out ways to alleviate stress.
F5. You can regulate your emotions effectively and remain unaffected by negative feelings.

The strikethrough indicates that this item was excluded based on the subsequent confirmatory factor analysis and, therefore, was not included in the final version of the Adolescent Health Behavior Checklist.

**Table 2:** Factor structure of the exploratory Adolescent Health Behavior Checklist.

**Item**	**Factor A**	**Factor F**	**Factor D**	**Factor E**	**Factor B**	**Factor C**	***h* ^2^**
A1	0.66						0.61
A2	0.78						0.74
A3	0.80						0.74
A4	0.78						0.68
A5	0.64						0.56
A6	0.67						0.66
B1					0.42		0.58
B2					0.54		0.52
B3					0.74		0.62
B4					0.62		0.61
B5					0.49		0.58
B6					0.47		0.69
C1						0.71	0.59
C2						0.73	0.59
C3						0.45	0.61
C4						0.42	0.56
C5						0.51	0.61
C6						0.58	0.63
D1			0.72				0.72
D2			0.57				0.65
D3			0.78				0.80
D4			0.66				0.73
D5			0.68				0.70
E1				0.74			0.72
E2				0.62			0.55
E3				0.68			0.62
E4				0.78			0.72
E5				0.58			0.65
E6				0.40			0.48
F1		0.62					0.62
F2		0.67					0.67
F3		0.62					0.65
F4		0.67					0.64
F5		0.61					0.77

*h*^2^, communality.

**Table 3:** Cronbach's alpha of the Adolescent Health Behavior Checklist.

**Factor**	**EFA (*N* = 177)**	**CFA (*N* = 349)**
Exercise	0.89	0.89
Diet	0.78	0.80
Personal responsibility	0.88	0.79
Sleep	0.88	0.88
Interpersonal relationships	0.87	0.84
Stress management	0.87	0.87
Global	0.96	0.95

EFA, exploratory factor analysis; CFA, confirmatory factor analysis.

### 3.2 Confirmatory factor analysis

**Figure 3:**
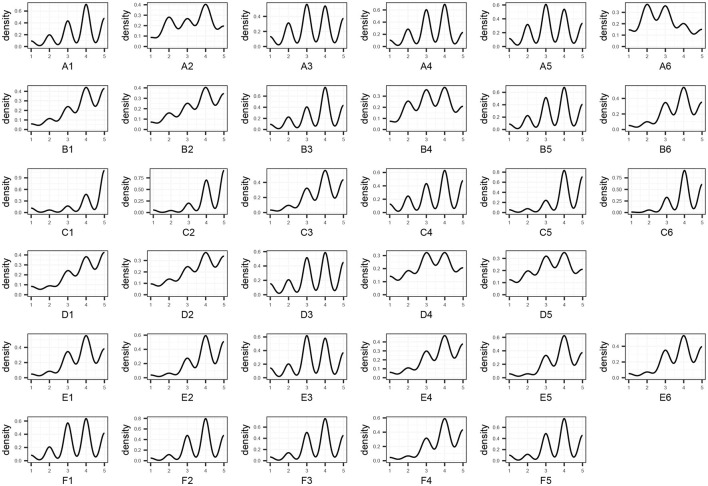
Item-level data of the confirmatory Adolescent Health Behavior Checklist.

For the draft 34-item AHBC, it was estimated that 340 participants would be needed for confirmatory factor analysis. A total of 400 questionnaires were distributed, and 349 valid questionnaires were used for the analysis. Figure 3 shows the data structure from the 34 items. In brief, the nature of an ordinal Likert scale precludes multivariate normality of questionnaire responses, which justifies the distributional free analytic approach.

**Figure 4:**
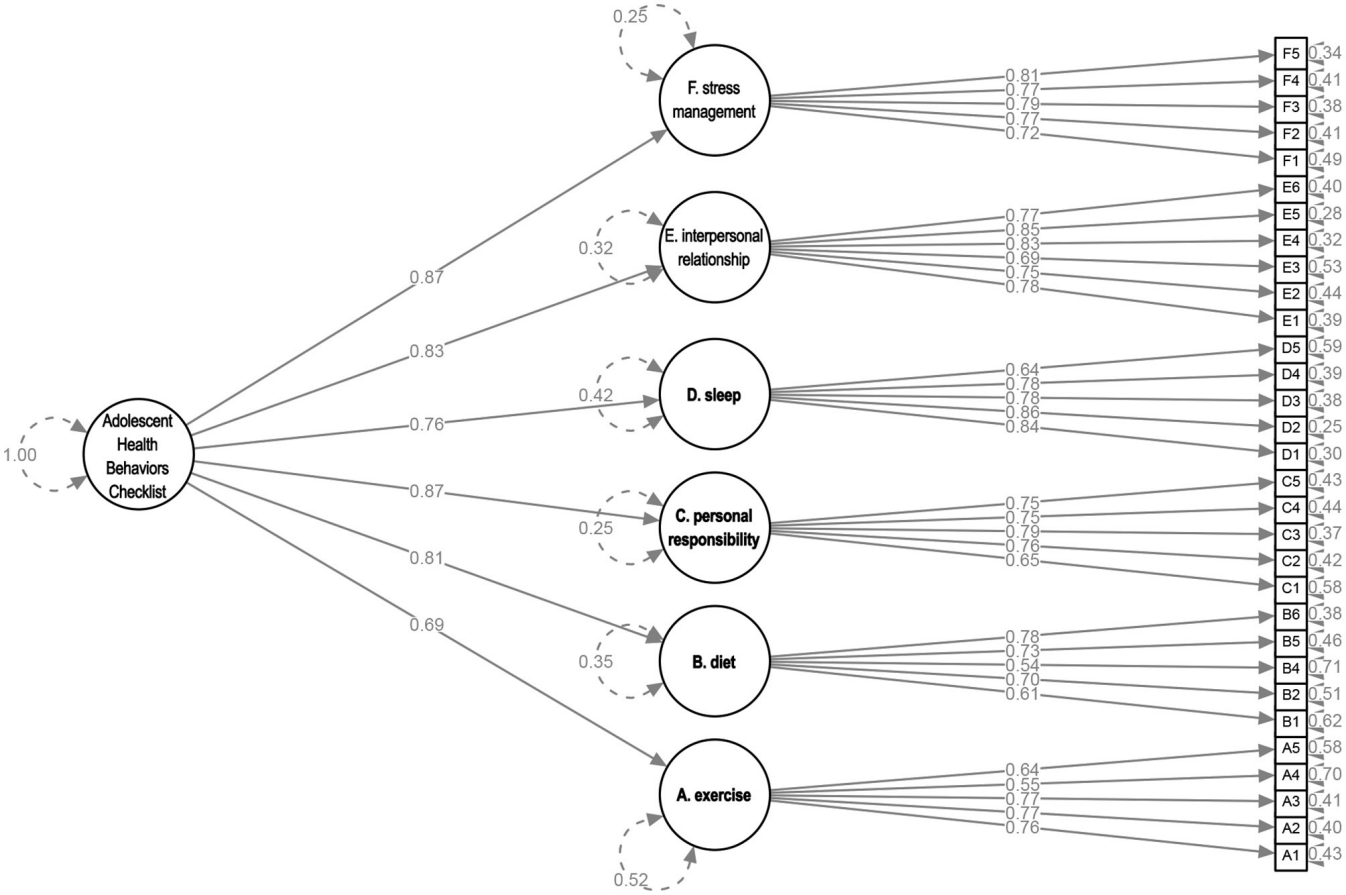
Confirmatory factor analysis of the Adolescent Health Behavior Checklist in relation to a 6-factor model with a global health behaviors factor. The values in the figure are standardized coefficients, with residual errors depicted inside circles.

After fitting the full dataset, the standardized factor loadings of items A6, B3, and C6 fell below 0.50, leading to their removal from the AHBC. The refitted AHBC yielded an SRMR of 0.058, indicating adequate model fit. Additionally, the CFI of the measurement model was 0.990, meeting the traditional cutoff criteria (31). Based on these absolute and comparative fit indices, it was concluded that the 31-item AHBC demonstrates acceptable factorial validity, as shown in Figure 4.

To ensure the convergent validity of the constructs, we assessed McDonald's omega and the AVE for each construct, as shown in Table 4. While the diet factor slightly fell below 0.8, indicating a slight deviation, all other factors exhibited composite reliability exceeding 0.8. This suggests that a significant portion of the factor variance was attributed to true score variance. The factor loading of the exercise was approximately 0.7, and the remaining five factor loadings surpassed 0.7. Although the AVE for the diet factor was below the recommended benchmark value of 0.5, the convergent validity remains adequate since all composite reliabilities were above 0.6 (34). Thus, the convergent validity of the designated construct is considered acceptable.

**Table 4:** McDonald's omega (ω) and average variance extracted (AVE) of the 31-item Adolescent Health Behavior Checklist.

	**Exercise**	**Diet**	**Personal responsibility**	**Sleep**	**Interpersonal relationships**	**Stress management**
ω	0.81	0.79	0.82	0.89	0.89	0.86
AVE	0.50	0.46	0.55	0.62	0.61	0.59

We concluded the factor analysis by examining the discriminant validity and internal consistency of the AHBC. The estimated factor correlations were all below the cutoff value of 0.8, and the significant chi-squared statistic presented in Table 5 further supported discriminant validity. Additionally, the Cronbach's alpha values, detailed in Table 3, exceeded 0.7 for all factors. Taken together, these findings confirm that the 31-item AHBC demonstrates acceptable construct validity. The Chinese-language AHBC (2024 version) has been designed (DOI: m9.figshare.27315609.v1).

**Table 5:** Estimated factor correlations of the 31-item Adolescent Health Behavior Checklist.

**Pair**	**CI_CFA_ (sys)**	χ^**2**^ **(sys)**		
	**Upper 95%**	ρ_CFA_	χ^2^ **difference**	***p*** **(**χ^2^**)**
A ~~ B	0.66	0.62	53.2	< 0.001
A ~~ C	0.61	0.57	99.2	< 0.001
A ~~ D	0.57	0.53	163.2	< 0.001
A ~~ E	0.56	0.52	194.0	< 0.001
A ~~ F	0.67	0.63	64.5	< 0.001
B ~~ C	0.74	0.70	19.2	< 0.001
B ~~ D	0.66	0.62	64.7	< 0.001
B ~~ E	0.68	0.65	57.5	< 0.001
B ~~ F	0.72	0.68	33.5	< 0.001
C ~~ D	0.66	0.63	78.4	< 0.001
C ~~ E	0.79	0.76	5.1	0.023
C ~~ F	0.78	0.74	10.0	0.002
D ~~ E	0.66	0.63	118.1	< 0.001
D ~~ F	0.71	0.68	51.8	< 0.001
E ~~ F	0.74	0.72	30.7	< 0.001

A, exercise; B, diet; C, personal responsibility; D, sleep; E, interpersonal relationships; F, stress management.

## 4 Discussion

In China, there are well-established national standards for measuring the physical development and fitness of adolescents. However, there is no suitable health behavior assessment tool for adolescents due to the complex nature of human behaviors, different value orientations, and reliance on subjective self-evaluation. As a result, policymakers do not have an appropriate instrument to assess the effectiveness of relevant school policies. To address this gap, this study designed a 31-item AHBC, which demonstrated good model fit and construct validity through exploratory factor analysis and confirmatory factor analysis. From the perspective of the rating methodology (see below), the AHBC can be viewed as a universal guideline to enhance adolescents' health literacy and act as a preliminary step toward improving health behaviors (35). Therefore, this instrument can be used to assess students' health behaviors regularly and provide a scientific basis for policymakers, which is of great practical value.

Furthermore, this study makes three theoretical contributions to the existing literature. First, from a strictly statistical perspective, the AHBC exhibits the highest level of construct validity among similar instruments. The CFI of the AHBC reached 0.990, surpassing existing Chinese-language instruments (11, 21). Additionally, both the factor loadings of the AHBC's six latent variables and individual items were higher than 0.5, with 81% of the item-level factor loadings exceeding 0.7. In comparison, the 5-factor Chinese version of the Health-Promoting Lifestyle Profile-II had only 37% of item factor loadings above 0.7 (18), and the Chinese-language Adolescent Health Promotion scale short form had only 33% of item factor loadings above 0.7 (21). This demonstrates that the variance in observed variables can be better explained by the factor-item structure in the AHBC. Therefore, the AHBC has the best construct validity for rating adolescent health behaviors to date.

Second, this study remedies the shortcomings of existing instruments. Although the Health-Promoting Lifestyle Profile has been validated for adolescent samples, some items are not entirely appropriate for this population (18). For example, adolescents may not yet have the ability to question an “MD/second opinion” in the health responsibility dimension. Similarly, due to cultural differences, items such as spiritual growth and eating cheese are not applicable to Chinese adolescents. More importantly, the AHBC addresses a common shortcoming of current instruments: the lack of assessment of sleep behavior. Even in the most authoritative WHO instrument, only one of the 128 items asks about sleep quality (15). Realistically, the percentage of Chinese adolescents with sleep disorders is increasing quickly (36), and this phenomenon is also observed in other regions regardless of socioeconomic conditions (37). Sleep deprivation not only affects learning, leading to more academic stress and anxiety, but in the long run, it can have immeasurable negative effects on physical and mental wellbeing. Therefore, the dimensions covered by the AHBC are more contemporary and promote the development of a theoretically comprehensive assessment of adolescent health behaviors.

Third, the study was not limited by traditional scoring methods of structural equation models but instead utilized a more practical approach to evaluation. Similar instruments use a weighted total score for each dimension to rate adolescent health behaviors (10, 12). However, this approach is problematic both theoretically and practically. Theoretically, while weighted sum scoring may seem reasonable, all weighting methods have inherent flaws, making the approach questionable (38). Practically, weighted sum scoring can lead to nuances being overlooked and can result in misleading evaluations. Using our AHBC as an example, if Student XY scores three on each of the exercise items and five on each of the diet items, and Student YZ scores four on each of the exercise and diet items, the two students would have the same health behavior rating if calculated as a total score. However, in practice, it is clear that Student XY has significant deficits in the exercise behaviors. Therefore, the weighted sum scoring approach does not accurately reflect an individual's overall level of health behaviors. To address this, we considered this issue in the initial design of the AHBC, opting for a checklist format rather than a scale. We believe that all six dimensions of the assessment are equally important. When students self-assess, if the score of a single item is lower than four, it indicates that the behavior needs improvement. This approach aligns with the theory that good health behaviors are indispensable and adheres to the principle that a checklist is used to identify and solve problems in practice. For policymakers, we recommend using mean scores for cross-sectional (e.g., school-to-school, province-to-province) and longitudinal comparisons, with four as the baseline passing score. While this method is not perfect, it is highly operational. The AHBC can continue to facilitate cross-sectional and longitudinal comparisons as amended or new items are added.

This study also has several limitations. First, due to financial and time constraints, we only verified the construct validity in a middle school student population. Future research needs to conduct further cross-validation with high school students. Second, the sample for this study is limited to the Tianjin region, which not only has superior economic development but also has very high educational attainment among the 31 administrative regions in China. Therefore, future research should expand the study to other regions for cross-validation and, theoretically, conduct factorial invariance validation for different levels of socioeconomic conditions and gender. Third, we invited experts from Tianjin University of Sports and Shaanxi Normal University to participate in the Delphi method. However, none of the invited experts specialized in the fields of nutrition or sleep science. For example, item B4 argues that a high salt diet is not good for health. For adolescents, consumption of a diet high in sugar might be more harmful to health than a diet high in salt (39). Therefore, although this AHBC has construct validity, we believe that some items can be further optimized to more scientifically and accurately assess adolescents' health behaviors in modern life.

We suggest that future research should improve the scientific quality and practicality of the AHBC. To enhance the AHBC, the WHO or an expert group from China's Ministry of Education should engage specialists in relevant fields to refine and expand the items of each of the six dimensions. The aim is to create multilingual versions of the AHBC that cater to various populations. For example, the WHO commissioned Global Action for the Measurement of Adolescent Health Advisory Group consists of a culturally and scientifically diverse panel of experts (40), which could theoretically offer stronger evidence-based recommendations for a comprehensive health behavior checklist. Additionally, in China, it is recommended that the Ministry of Education develops a mobile application that will regularly collect data on students' health behavior ratings. Utilizing electronic storage and feedback systems, policy priorities and individual behavioral patterns can be promptly adjusted, leading to the enhancement of adolescents' healthy behavioral patterns through scientific approaches. Finally, we encourage other scholars to conduct additional measures of construct validity. For instance, concurrent validity could be assessed by comparing the checklist with other health-promotion scales (18, 19). Researchers could also creatively validate certain items using externally derived, objective measures, such as examining the relationship between exercise behavior (item A3) and actigraphy-based movement data, or between sleep behavior and actigraphy-based sleep data. Similarly, predictive validity could be tested by evaluating whether poor scores—such as those related to sleep patterns and stress management—translate into clinically meaningful outcomes (e.g., insomnia) or impact academic performance. More rigorous testing is necessary to enhance the validity of the AHBC before it can be widely implemented in school settings.

In conclusion, this study fills a gap in assessing health behaviors among Chinese adolescents. The hierarchical, 6-factor AHBC's comprehensive assessment dimensions and practical evaluation approach contribute to advancing health literacy theories in this field. With further validation and improvement, this tool has the potential to effectively assist adolescents in measuring their all-around health behaviors. This will be beneficial for shaping policies and positively influencing the development of society.

## Data Availability

The raw data supporting the conclusions of this article will be made available by the authors, without undue reservation.
